# Physiological Data Integration and Predictive Modeling in Intensive Care

**DOI:** 10.3390/life16081254

**Published:** 2026-07-29

**Authors:** Bianca Liana Grigorescu, Leonard Azamfirei, Sânziana Bora, Dorin Bica, Irina Săplăcan, Raduly Gergo, Mihaly Veres

**Affiliations:** 1 Department of Anesthesiology and Intensive Care, George Emil Palade University of Medicine, Pharmacy, Science and Technology of Targu Mures, 540142 Targu Mures, Romania; 2 Department of Anesthesiology and Intensive Care Medicine, Emergency County Hospital, 540136 Targu Mures, Romaniagergo.raduly@umfst.ro (R.G.); 3 Doctoral School of Medicine and Pharmacy, George Emil Palade University of Medicine, Pharmacy, Science and Technology of Targu Mures, 540139 Targu Mures, Romania; 4 Faculty of Engineering and Information Technology, George Emil Palade University of Medicine, Pharmacy, Science and Technology of Targu Mures, 540142 Targu Mures, Romania; 5 Department of Anatomy and Embryology, George Emil Palade University of Medicine, Pharmacy, Science and Technology of Targu Mures, 540139 Targu Mures, Romania; 6 Department of Anesthesiology and Intensive Care, Emergency Institute for Cardiovascular Diseases and Transplantation, 540136 Targu Mures, Romania

**Keywords:** intensive care unit, Smart ICU, clinical decision support, hemodynamic instability, delirium ICU, AI prognostic tools, machine learning, artificial intelligence

## Abstract

Intensive care medicine represents one of the most challenging setting in modern healthcare, where specific mechanisms intertwine and form a dynamic biological model, where organ dysfunction can easily evolve to multi-organ dysfunction, continuously reshaping the patient’s clinical course. The critically ill patient represents a biological system resulted from interaction between maladaptive and adaptative mechanisms, therefore generates a large volume of data that can exceeds human cognitive capacity. Artificial intelligence can integrate multimodal physiological, laboratory, and clinical data into a dynamic representation of the patient’s biological trajectory. AI-tools and machine learning technologies have evolved to potential clinical support tools, with great perspectives for future implementation, but currently with limited use in clinical practice. This article is a narrative review of artificial intelligence in ICU, aiming to present current evidence and limitations.

## 1. Introduction

Intensive care medicine represents one of the most demanding setting in modern healthcare because the clinical condition of critically ill patients can change continuously and rapidly over time. Unlike most medical specialties, where diseases predominantly affect a single organ system, critically ill patients simultaneously develop multiple interacting pathophysiological processes. Systemic inflammation, immune dysregulation, hemodynamic instability, respiratory failure, metabolic disturbances, neurological dysfunction, endothelial injury coexist, and organ dysfunction often leads to progressive multi-organ failure. These mechanisms intertwine and form a dynamic biological model, where organ dysfunction can easily evolve to multi-organ dysfunction, continuously reshaping the patient’s clinical course.

Therefore, the critically ill patient represents a biological system resulted from interaction between maladaptive and adaptative mechanisms, in response to organ dysfunctions. The ICU patients constitute an extremely heterogeneous population, due to the pre-existing comorbidities, underlying disease, physiological reserve and therapeutic requirements, resulting in markedly individualized clinical trajectories [1].

From another perspective, ICU can be defined as a dynamic environment in which pathology-related mechanisms, therapeutic interventions and host response mechanisms continuously interact [2,3].

In the critically ill patient, the systemic inflammatory syndrome, endothelial dysfunction, organ dysfunction and neurocognitive impairment are overlapping, making it difficult to identify specific changes associated with a particular pathology, as such alterations often precede laboratory or imaging modifications—thereby potentially delaying prompt and effective decision-making. Real-time Artificial Intelligence (AI) analysis of monitoring curves and specific parameters can draw attention to early changes that a clinician might otherwise overlook.

Modern intensive care units generate a large volume of data from continuous monitoring, biomarkers and laboratory data, imaging, mechanical ventilation, organ-specific support therapy, medications, infusion pumps, bedside assessments, and electronic health records [4]. The temporal complexity of these data often exceeds the capacity of continuous human cognitive integration.

Artificial intelligence has emerged with the purpose of integrating a large volume of data to identify patterns, phenotypes, and predictive analysis for specific pathologies, such as sepsis, hemodynamic degradation, organ-specific dysfunction–acute renal failure, respiratory dysfunction, neurological deterioration. By generating patterns and predictive scores, it can identify earlier signs of decompensation, therefore allowing early therapeutic interventions. Rather than replacing clinical reasoning, AI should be used as a decision-support tool for the clinician.

Accordingly, this narrative review evaluates the potential role of AI to predict the clinical course of critical illness through integration of multimodal physiological data. It also discusses the methodological limitations and implementation challenges encountered in clinical practice, and outlines future directions towards physiology-driven critical care.

## 2. Materials and Methods

This article is a narrative review of artificial intelligence in ICU, aiming to present current evidence and limitations. We performed a database search on PubMed/MEDLINE, Web of Science, with no minimum publication date limitation, using the following key-words: “intensive care unit”, “Smart ICU”, “clinical decision support”, “sepsis”, “septic shock”, “hemodynamic instability”, “delirium ICU”, “AI prognostic tools”, “machine learning”, “artificial intelligence”, “ patient data management system”.

Original articles, observational studies, systematic reviews and meta-analyses published in English with relevance to at least one of the central themes of the review were included. Articles focused on non-critical fields were excluded. The literature was selected through an expert-driven process; article selection was performed by screening the title and abstract, followed by full-text reading for relevant papers and manually selection of relevant references from the keys articles found, to identify additional studies that would augment our research. The primary goal of this article was not to perform a quantitative synthesis of evidence, typically performed by systematic reviews, but to integrate current knowledge on AI models potential and current limitations into a comprehensive framework.

## 3. Discussion

### 3.1. Understanding the Complexity of Critical Illness

Critically ill patients generate multiple physiological signals that individually describe organ function, but collectively describe the biological trajectory of critical illness.

The physiological deterioration rarely occurs abruptly. Instead, critically ill patients usually progress through a phase of subtle biological instability that precedes manifest clinical decompensation. Similar to other biological systems, critical illness may undergo abrupt transitions after reaching critical tipping points, shifting from compensated physiology toward rapid multiorgan dysfunction [5]. Importantly, these transitions are frequently preceded by discrete physiological alterations that are below the detection threshold of conventional monitoring systems.

These early stages of instability represent a potentially reversible therapeutic window, commonly referred as the “golden hours” of critical illness. Initially described in trauma, this concept was extended to cardiac emergencies, neurological emergencies and lately, sepsis and septic shock. Timely recognition leads to timely intervention, and therefore, improves outcomes by reducing the progression to irreversible damage [6].

#### 3.1.1. Why Does Physiology Need Integration?

Regardless of the underlying etiology, critically ill patients present remarkably similar manifestations-hypotension, tachycardia, altered mental status, hyperlactatemia, hypoxemia, oliguria, and systemic inflammatory responses, despite having different underlying mechanisms. Consequently, distinct diseases may simultaneously contribute to the same pattern of physiological deterioration. The intensivist is demanded not only to recognize organ dysfunction but, more importantly, to identify its predominant biological trigger while the disease process remains potentially reversible.

Continuous physiological monitoring provides a real-time representation of organ function. Moreover, there are no individual physiological variable that adequately reflects the biological response of critical illness. Cardiovascular, respiratory, metabolic, neurological, and inflammation ongoing interact, resulting in physiological patterns that cannot be solely interpreted.

#### 3.1.2. Multimodal Integration of Physiological Data

• Data acquisition: Critically ill patients generate a diverse spectrum of datasets, being characterized by fluctuating evolution and sudden changes. Multimodal integration requires data generated by patients vitals, bedside monitors–heart rate, ECG waveforms, mean arterial pressure, heart rate variability, oxygen saturation; invasive and non-invasive monitoring systems, ventilatory parameters from ventilators, medications used and doses from infusion pumps, laboratory and imaging data from EHR, and nursing documentation.
• Temporal synchronization: Data acquisition does not record all physiological variables at the same time or the same frequency, therefore temporal synchronization is essential in order to align heterogenous data onto a common timeline, to preserve the dynamic relationship and to recognize the evolving trajectory of deterioration, improving the model’s predictive capacity.
• Preprocessing: improves data quality before model development, by artifact removal, noise filtering and handling missing data. This step is essential for increasing the reliability of the AI model.
• Feature extraction: converts raw physiological data into variables that can be analyzed by ML algorithms, enabling AI models to recognize subtle changes before clinical deterioration becomes obvious.
• Mutimodal fusion requires integration of multiple physiological variables and generates a representation of the patient’s biological status. The multimodal fusion is essential for an AI model to analyze and lean the continuous interactions between the collected signals, allowing the model to recognize the relationship between them, reflecting the complexity of critical illness and the evolving state of the critical patient.
• Dynamic temporal modeling: allows AI algorithms to learn how physiological data change and interact over time and identify trends that may precede clinical deterioration. This approach is particularly relevant in intensive care, emphasizing the dynamic process of critical illness.

**Figure 1:**
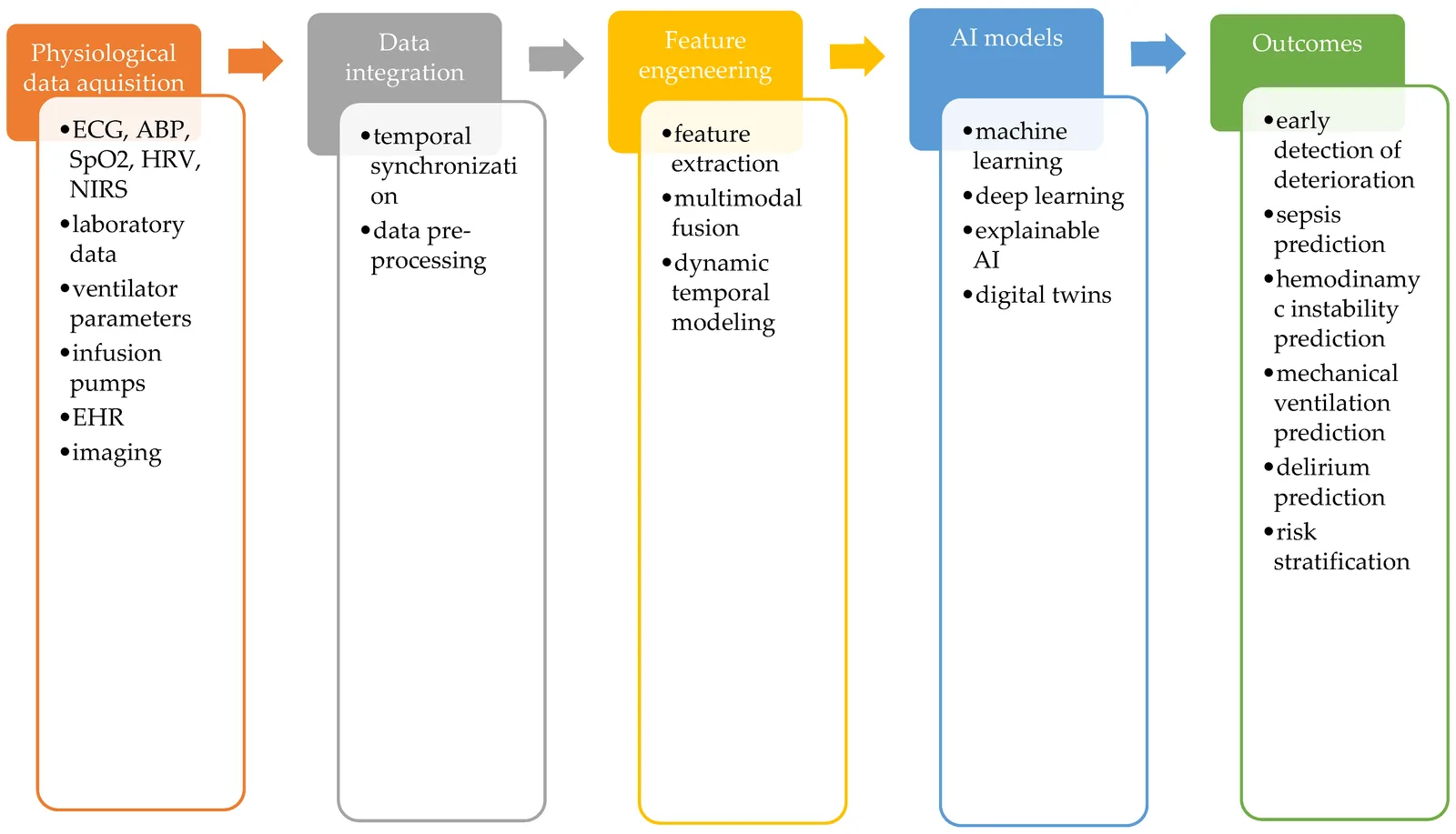
Multimodal integration of physiological data in AI models.

Figure 1 summarizes the process of multimodal integration of physiological data and integrates the central concepts discussed throughout this review.

#### 3.1.3. Why Are Conventional Prognostic Scores No Longer Sufficient?

To improve risk stratification, prognostic scoring systems such as Sequential Organ Failure Assessment (SOFA) and Acute Physiology and Chronic Health Evaluation (APACHE) II, APACHE III or APACHE IV have become essential in intensive care practice. These scores estimate illness severity using physiological variables, and have demonstrated considerable value for mortality prediction, performance evaluation, and clinical research. However, their principal limitation resides in their static nature. Because critically ill patients experience continuous physiological changes, a single baseline assessment frequently fails to reflect the evolution of critical illness [7].

In contrast, dynamic prognostic models that incorporate laboratory results, bedside physiological measurements, treatment interventions, and electronic health record data can provide a more accurate representation of the patient’s course. In sepsis, although the SOFA score remains the most used indicator of organ dysfunction, longitudinal data monitoring has shown better results concerning risk of deterioration [8,9]. Similarly, APACHE II and Simplified Acute Physiology Score (SAPS) II, commonly used in ICU, are static representations of patient’s status at a certain moment. Consequently, increasing attention has shifted toward dynamic prediction models capable of continuously updating individual patient risk during the ICU stay [10,11].

### 3.2. Multi-Monitoring the Critically Ill Patient

#### 3.2.1. Why Is Continuous Physiological Monitoring Fundamental in Critical Care?

Continuous physiological monitoring represents the cornerstone of modern intensive care medicine because critically ill patients may deteriorate rapidly, often before evident clinical manifestations become apparent.

The clinical value of real-time monitoring extends beyond the observation of individual physiological variables. The interpretation of continuously acquired physiological data requires integration rather than isolated analysis. Artificial intelligence aims to enhance recognition of specific patterns of deterioration, in [12,13].

#### 3.2.2. Can Cardiac Electrical Activity Monitoring Reveal Early Physiological Deterioration?

Electrocardiogram (ECG) is one of the main non-invasive sources of information regarding cardiovascular events, reflecting autonomic nervous system activity, myocardial function and hemodynamic state of the critically ill patient. By extracting data from ECG signals, AI algorithms showed promising results by reducing false arrythmia alarms, recognizing left ventricular dysfunction and early signs of hemodynamic decompensation, and predicting one-year mortality among cardiac ICU patients [12,14]. Using MIMIC-III data base, Mollura et al. created a closed-loop cardiovascular AI model derived from continuously recorded vital waveforms of heart rate, blood pressure and heart rate variability, along with data extracted from EHR, that was capable to identify sepsis within 1 h after admission in the ICU, achieving an AUROC of 0.92 and AUPRC of 0.90. They created a representation of a potential prospective monitoring system, paving the way for the development of higher level algorithms for future studies [12].

Rather than relying on fixed heart-rate values, AI interprets dynamic changes in cardiac rhythm and heart rate variability (HRV). Because HRV reflects autonomic regulation, it has emerged as an important physiological marker for the early identification of sepsis, prediction of delirium, and optimization of sedation strategies [13].

#### 3.2.3. Why Is Arterial Pressure More than a Blood Pressure Measurement?

Mean arterial pressure (MAP) remains the principal bedside indicator of organ perfusion pressure and therefore represents a cornerstone of hemodynamic monitoring. Tissue perfusion, a derivate parameter, depends on the response of arterial blood pressure, cardiac output, vascular tone, venous pressure, and microcirculation. Consequently, identical MAP values may correspond to markedly different physiological states among critically ill patients.

By analyzing the dynamic interaction between cardiac performance and arterial pressure responses, waveform-based models have demonstrated the ability to identify sepsis within the first hour following ICU admission [12,15]. Furthermore, because MAP contributes to cerebral perfusion pressure, continuous integration of arterial pressure waveforms with cerebral blood flow monitoring may facilitate identification of the lower limit of cerebral autoregulation, thereby supporting individualized cerebral perfusion management [16].

#### 3.2.4. Can Continuous Monitoring Characterize Tissue Perfusion?

Hemodynamic coherence cannot be assessed solely through macro-circulatory variables. Parameters reflecting tissue oxygen delivery and utilization remain essential of cardiovascular function. The venous-to-arterial carbon dioxide partial pressure difference (PvaCO_2_ gap) has emerged as a valuable bedside marker of cardiac output performance and tissue perfusion. In patients with sepsis, increased CO_2_ gaps may also reflect cytopathic hypoxia, allowing clinicians to differentiate between macro-circulatory stabilization and persistent microcirculatory dysfunction [15].

Similarly, neural networks and other deep learning architectures reported efficient analysis of oxygenation variables, including arterial oxygen saturation (SpO_2_) and regional cerebral oxygenation assessed by near-infrared spectroscopy (NIRS), reflecting the cerebral oxygen delivery capacity [14,16].

#### 3.2.5. Can Cardiovascular Monitoring Anticipate Hemodynamic Deterioration?

Hemodynamic instability represents one of the most life-threatening conditions encountered in the intensive care unit, usually leading to multiple organ dysfunction [17,18]. The physiopathology behind this syndrome is explained by alteration in myocardial contractility, vascular tone, autonomic regulation, and microcirculatory function. Timely intervention requires recognizing the therapeutic window before clinical manifestation–hypotension and low cardiac output-occur.

Although conventional parameters remain indispensable in daily practice, they provide only a partial representation of an evolving physiological process and therefore have limited ability to anticipate hemodynamic deterioration before shock becomes clinically evident [19,20].

Machine-learning models like Time-varying Hemodynamic Early Warning Score (TvHEWS) and Hemodynamic Stability Index (HSI) have demonstrated the ability to predict hemodynamic instability up to 24 h prior it becomes clinically obvious, vasopressor requirements and fluid resuscitation [19,20]. In addition, a neural network (Physiological Deep Learning—PDL model) developed by Cherifa et al. could predict the evolution of mean arterial pressure (MAP) and heart rate values up to 60-min ahead of time. By using a multi-task learning (MLT) architecture that analyzes HR and MAP, it showed better results than most predictive models that used only MAP signal. Their model reached a predictive value of 90% for acute hypotensive episodes in patients at high risk (predicted MAP < 60 mmHg). Although limited by the lack of prospective validation, their results are supporting the use of a deep learning model with multitask learning structure and it’s potential to help clinicians [17].

Together, these studies describing AI models suggest a shift in perspective, from detecting hypotension to predicting the risk of hemodynamic alteration that led to circulatory failure, anticipating hemodynamic instability before clinically obvious hypotension develops.

Cardiovascular assessment extends beyond macro-circulation. Tissue perfusion reflects oxygen delivery; therefore, the venous-to-arterial carbon dioxide partial pressure difference (PvaCO_2_ gap) and regional cerebral oxygenation can serve as the bed-side indicators of microcirculatory dysfunction, and indirectly of cardiac output [18].

#### 3.2.6. Can AI Recognize Respiratory Deterioration Before Respiratory Failure Becomes Clinically Evident?

Acute respiratory failure remains one of the most common pathology encountered in ICUs. Although mechanical ventilation is a life-saving intervention, ventilator-induced lung injury, ventilator-associated pneumonia and the difficult process of weaning lead to prolonged ICU stay, and increase the mortality rates [21]. Identifying patients approaching respiratory decompensation before overt respiratory failure therefore represents another major challenge in critical care medicine.

Conventional clinical scores, including Modified Early Warning Score (MEWS), quick SOFA, and the ROX index, summarize respiratory status using a limited number of physiological variables and consequently demonstrate only moderate predictive performance [21].

Recent AI-based prediction models have substantially expanded this perspective, showing promising results predicting the need for mechanical ventilation, based on respiratory parameters, 24 h prior it becomes necessary. Prediction accuracy was improved by worsening respiratory parameters. Shashiku et al.’s deep learning algorithm predicted mechanical ventilation requirement 24 h before initiation, with an AUC of 0.918–0.943, but the study is limited by the lack of generalizability and the need for further external validation [21]. Deep learning-analysis of thoracic X-rays, by monitoring the subtle radiographic changes several days before respiratory degradation was clinically evident, may improve prediction. The study on this manner conducted by Kulkarni et al. achieved 90% accuracy, 86% sensitivity, and 84% specificity, outperforming clinical judgment, but it was limited by the single-center design and COVID-19 limited population [22].

#### 3.2.7. Why Do Laboratory Biomarkers Remain Indispensable?

Laboratory investigations remain fundamental for characterizing the biological mechanisms underlying critical illness. For this reason, AI models combine continuously monitored physiological signals with metabolic variables, inflammatory, cardiac specific-troponins, natriuretic peptides, and neurological biomarkers-glial fibrillary acidic protein (GFAP), S-100B protein or neuron-specific enolase, to enhance the assessment of organ dysfunction, risk stratification, phenotyping and support individualized therapeutic strategies [12,13,15,16,23].

Blood gas analysis represents another cornerstone of physiological assessment, allowing easy bed-side evaluation of respiratory function, acid-base status and indirectly, cardiac and respiratory interactions. AI systems increasingly combine these laboratory measurements with continuously recorded physiological waveforms, allowing assessment of patient’ status during intervals in which laboratory data are unavailable [12,15].

#### 3.2.8. Can Clinical Context Improve Physiological Interpretation?

Continuous physiological signals cannot be interpreted independently of clinical context. Medication exposure, specifically vasopressors and sedative administration, can be used for prediction of sepsis progression [12,23]. Similarly, AI-assisted analysis of electroencephalographic patterns can assess sedation depth and delirium prediction, analysis of respiratory waveforms from the ventilator can facilitate ventilator desynchrony prediction, can estimate degradation in respiratory status and assess the possibility for weaning from the ventilator [13,14]. Hemodynamic management may also benefit from AI-assisted interpretation, supporting an individualized management regarding vasopressor and inotropic requirements, or influence the fluid resuscitation strategies [15]. Finally, integrating nursing observations into the overall assessment of the patient facilitates timely preventive interventions [14].

### 3.3. Sepsis and Septic Shock—A Continuous Critical Challenge in ICU

Sepsis remains a leading cause of mortality among critically ill patients. Although major advances have been achieved in intensive care medicine, early recognition remains challenging because the initial manifestations are nonspecific, and may occur in different pathologies.

The current Sepsis-3 definition describes sepsis as life-threatening organ dysfunction caused by a dysregulated host response to infection [24]. This change in paradigm regarding sepsis is shifting the perspective regarding understanding the underlying mechanisms and influence the management of the septic patient. Nevertheless, despite improved understanding of sepsis pathophysiology and advances in supportive care, sepsis continues to account for substantial morbidity and mortality among critically ill patients [12].

#### 3.3.1. Why Is Early Recognition of Sepsis So Difficult?

One of the principal challenges in diagnosing sepsis is that its earliest manifestations are rarely disease-specific. Tachycardia, hypotension, tachypnea, altered mental status, hyperlactatemia, fever, oliguria, or inflammatory biomarkers may reflect a broad spectrum of pathological conditions encountered in critically ill patients. These pathological variables are the result of overlapping physiological disturbances caused by an inappropriate response of the host, causing systemic inflammation, cardiovascular collapse, metabolic stress, hormonal changes and tissue hypoperfusion. The intensivist must understand the difference between compensatory mechanisms and early signs of organ dysfunction, mainly because this is the narrow therapeutic window where clinical manifestations are reversible and the therapeutic intervention can be effective.

#### 3.3.2. Why Are Conventional Diagnostic Approaches Insufficient?

Early identification of sepsis is currently based on laboratory results and microbiological testing. Although they remain fundamental components in clinical practice, they are limited by the temporal delay between the onset, time of admission, sample acquisition, laboratory processing, and clinical interpretation.

While valuable for assessing illness severity and estimating prognosis, the commonly used scores-including SOFA, National Early Warning Score (NEWS), Modified Early Warning Score (MEWS) and SAPS II, are less capable of recognizing the nonlinear physiological responses that characterize the earliest phases of sepsis [25].

#### 3.3.3. Why Can AI Improve Early Sepsis Recognition?

Several retrospective studies have demonstrated that machine learning models can predict sepsis 4–12 h before conventional scores used in the routine practice, enabling earlier therapeutic intervention, but they are limited by the retrospective design [25,26]. The study conducted by Wong et al. evaluated the performance of the Epic Sepsis Model (ESM), a sepsis prediction model implemented at hundreds of US hospitals. The external validation cohort study found that ESM has poor discrimination and calibration in predicting the onset of sepsis, with an AUC of 0.63, sensitivity of 33% and positive predictive value of 12%, a lower predictive accuracy than previously reported. This study raised fundamental concern regarding the need for independent validation of clinical AI tools before widespread implementation [27]. In contrast to the retrospective studies we have found, Adams et al. conducted a prospective study evaluating the association between prompt provider confirmation of an ML-based sepsis alert system (TREWS—Targeted Real-time Early Warning System) and patient outcomes, proving a reduced rate of in-hospital mortality, organ failure and length of stay, supporting the clinical benefit of AI-assisted early sepsis recognition [28].

#### 3.3.4. Can AI Characterize Cardiovascular Dysfunction in Septic Patients?

Systemic inflammation leads to multiple organ dysfunction, specifically sepsis-induced cardiac dysfunction (SICD). AI-based system may integrate echocardiographic findings (left ventricular longitudinal strain (LV-GLS), filling pressures, end-diastolic volume of the left ventricle, peak systolic strain), arterial pressure waveforms, tissue perfusion markers and laboratory biomarkers, enhancing the management of SICD and allowing continuous assessment of the disease progression [12,15].

The concepts of source control, antimicrobial empirical treatment, fluid therapy and vasoactive support were challenged by current guidelines, and the compliance to sepsis guideline recommendations is still poor [29,30]. Equally important concerning SICD is the timely adjustment of fluid therapy and vasoactive support, as physiological status may change rapidly during the course of critical illness.

#### 3.3.5. From Prediction to Physiological Understanding in the Septic Patient

An important advantage of machine learning lies in its ability to analyze continuously acquired physiological signals immediately after ICU admission, when laboratory and microbiological data are unavailable. Physiological waveforms and continuously monitored vital signs can provide an immediate source of data, and ML has the potential to identify patterns associated with clinical deterioration [12].

Sepsis illustrates one of the most important characteristics of critical illness: physiological deterioration rarely occurs abruptly. Organ dysfunction evolves progressively, and is the result of overlapping responses from inflammatory, cardiovascular, respiratory, neurological, and metabolic systems. The critically ill patient should be regarded as a dynamic biological system in which subtle changes and interactions between multiple physiological processes frequently precede overt organ dysfunction. The use of AI has the potential to identify early signs of organ dysfunction by integrating specific data into a multi-model, analytical tool [19,20].

#### 3.3.6. Beyond Isolated Variables: Integrating Physiology, Laboratory Biomarkers, and Clinical Context

Clinical context, including medication exposure, vasopressor administration, ventilatory parameters, sedation, renal replacement therapy, nutritional support, neurological examination, and nursing observations, represent essential inputs for contemporary AI systems. Integration of these multimodal data enables prediction of disease progression, individualized management, prevention of medication errors, and identification of patients at risk for adverse clinical events [17,18,19,20,21,22].

A multimodal AI-model used for precision monitoring and clinical decision support would include several parameters acquired from heterogenous data—ECG, analyzing waveforms, heart rate, conduction abnormalities and ST morphology to detect ischemia; HRV, derived from the ECG, continuous arterial pressure waveforms, to integrate pulse pressure, variability, detect early signs of hypotension; oxygen saturation and NIRS monitoring, to assess respiratory deterioration, oxygen extraction and tissue perfusion; medication used (sedatives, vasopressors) integrating doses, half-life, interactions and correlating them with hemodynamic and respiratory changes. These integrated data can be further used for risk prediction, analyze trends, deterioration detection, treatment response prediction, mortality estimation, and individualized therapy recommendations.

### 3.4. Delirium in the ICU

Neurological deterioration frequently develops in a subtle and heterogeneous manner. Altered consciousness, inattention, agitation, delayed awakening, and cognitive fluctuations may reflect delirium, sedation, metabolic encephalopathy, hypoxemia, sepsis-associated encephalopathy, stroke, or non-convulsive seizures. These overlapping clinical manifestations make early recognition particularly challenging despite continuous bedside observation.

Delirium should not be regarded as an isolated neurological complication but rather as the cerebral expression of systemic critical illness.

#### 3.4.1. Why Is Neurological Deterioration So Difficult to Recognize in Critically Ill Patients?

Neurological deterioration is the most common manifestation of brain dysfunction, but because it rarely presents as an isolated disorder, it represents one of the most challenging complications encountered in critically patients. Several risk factors play an important role in development of delirium, including systemic inflammation, endothelial dysfunction, impaired cerebral autoregulation, autonomic imbalance, metabolic disturbances, hypoxemia, sedative exposure, sleep disruption, and organ failure. The multifactorial pathophysiology can interfere with early recognition and proper management [31]. Neurological dysfunction frequently evolves through subtle alterations that may remain clinically unapparent. The most frequent type—hypoactive delirium, is often mistaken for residual sedation or encephalopathy. Studies have proposed different preventive measures, including pharmacological interventions, with promising results regarding the use of second generation antipsychotics in decreasing delirium incidence [32].

#### 3.4.2. Why Are Conventional Delirium Screening Tools Insufficient?

Routine delirium assessment relies on validated bedside instruments, particularly the Confusion Assessment Method for the ICU (CAM-ICU), the reference screening tool for mechanically ventilated and non-verbal critically ill patients. CAM-ICU has improved detection, but intermittent assessments may miss the earliest signs preceding cognitive dysfunction [33]. This limitation grew interest in continuous monitoring of the neurological changes before clinical delirium becomes evident [34].

#### 3.4.3. Can Continuously Monitored Physiological Signals Reveal the Earliest Stages of Delirium?

HRV has emerged as one of the most promising biomarkers of early neurological deterioration, reflecting autonomic nervous system regulation. The reduced autonomic adaptability often precedes clinical manifestations of delirium [35]. Quantitative EEG (Q-EEG) demonstrated specific changes in cortical activity associated with delirium, but is limited by the high susceptibility to artifacts and the need for specialized interpretation [36].

#### 3.4.4. How Can AI Improve Delirium Prediction?

Machine-learning models combine continuously monitored ECG, HRV, photoplethysmography (PPG), respiratory waveforms, laboratory investigations, medication exposure, sedation status, and electronic health record data to generate continuously updated estimates of delirium risk. The aim of AI-based and machine-learning models is to capture the fluctuating evolution and different stages of delirium, and by identifying subtle changes preceding the onset, to improve prediction and prevention [28,37]. Park et al. developed a ML model with good predictive accuracy in predicting delirium in ICU patients, with consistent performance across internal, temporal and external validation (AUROC 0.82/0.73/0.82), but limited by the retrospective design and the lack of generalizability [38].

### 3.5. Can Artificial Intelligence Truly Transform Critical Care?

Artificial intelligence has evolved from a research tool into a technology that supports clinical reasoning in critically ill patients. By improving predictive accuracy, it improves early recognition, diagnosis and management of clinical alterations in critically ill patients.

Table 1 summarizes data from the literature regarding predictive models, their clinical applications, outcomes, evaluation metrics and current data limitations.

#### 3.5.1. Challenges for Clinical Implementation of AI in the ICU

Current AI models developed and internally or externally validated show promising performance, yet they encounter several challenges when deployed in real-world clinical environments [39]. ML and deep learning algorithms use multilayered artificial networks, but remain “black-box” models, with limited transparency, and therefore gaining limited trust from clinicians. Explainable AI plays a crucial role in improving model interoperability and transparency, by identifying and explaining the variables behind the predictions [40]. The quality of the training data plays a major role in the predictive efficiency of an AI model, making them susceptible to algorithmic bias and reducing their generalizability. Rigorous external validation in geographically or temporally distinct cohorts is the most direct test of generalizability [41]. As mentioned before, the retrospective design of the studies found in the literature are the main limitation of implementing AI models into clinical practice, with only few studies undergoing external validation or prospective multicenter validation. The risk of bias, data security, liability gaps and ethical concerns regarding patient confidentiality need to be addressed, especially as AI relies on large datasets [42]. Finally, successful implementation requires, integration into clinical workflows without increasing alarm fatigue of negatively impacting clinician decision-making.

#### 3.5.2. From Prediction to Augmented Clinical Intelligence

Prediction alone does not necessarily translate into better clinical outcomes, because anticipating adverse events is not the main purpose of managing a critically ill patient. A better understanding of the course of a disease is what changes the outcome.

The conventional severity scores used in ICU, as APACHE, SAPS, SOFA and Mortality Probability Model (MPM) have played an essential role in risk stratification, but are limited by the static design. These scores are quantifying illness severity at a certain time, without capturing the rapidly evolving physiology of critical illness [43]. Studies based on longitudinal SOFA trajectories have shown that the evolution of organ dysfunction over time provides clinically meaningful information and may identify subgroups of septic patients with different risks and outcomes [8].

Artificial intelligence offers a framework for this transition. By integrating data from monitors, infusion pumps and ventilators, imaging, electronic health records, AI may identify relevant patterns that are missed by the conventional assessment [4,44].

However, the future of AI is not to replace clinical judgment, but to enhance it by understanding the multiple interactions between inflammation, immune and metabolic dysregulation, hemodynamic instability, respiratory failure, neurological dysfunction [45]. This perspective is highly relevant for intensive care, where the patient cannot be reduced to a single variable, score or disease label.

Implementation of AI in clinical practice remains limited by risk of bias, clinicians’ acceptance, infrastructure and costs, but mostly by the lack of prospective validation [4].

Digital twins represent one of the most promising future directions in this field. A medical digital twin is a virtual representation of an individual patient that continuously integrates clinical, physiological, laboratory and imaging data, creating a virtual representation of a physical system used to simulate disease progression, predict treatment response and support personalized decision-making [46]. This concept allows integration of specific changes in cardiovascular, respiratory, neurological, immunological and inflammatory parameters, into a dynamic model. Although medical digital twins remain in an early stage of development, they represent a conceptual shift from prognosis toward treatment simulation and precision critical care, by enabling high-fidelity modeling of individual patients and allowing predictive modeling that facilitates early intervention.

The core concept of digital twin is patient-specific physiological simulation, capable of presenting the patient’s current physiological state by integrating cardiovascular, respiratory, neurological, renal, metabolic and immunological data into a unified framework. By integrating data from bed-side monitors, ventilators, infusion pumps along with EHR data, digital twin technology would reflect the real-time trajectory of the patient and the disease. Current evidence proved that by integrating data regarding blood glucose, nutrition, sleep patterns and physical activity, digital twin models were capable to create a patient-specific replica and predict postprandial glucose levels [47]. Digital twin technologies are applicable in various domains, including chronic disease management, surgical planning, rehabilitation, cardiology, oncology and genomics [48,49]. Although developed outside the intensive care, in critical care, this patient-specific replica can be used for forecast of physiological behavior, treatment responses, and disease tracking [49,50]. Specific applications would include assessing the hemodynamic response to fluid resuscitation and vasopressors initiation or dose adjustments, clinical response to changes on ventilatory parameters for patients with ARDS or other forms of respiratory failure, reducing the support and spontaneous breathing trials for weaning tentative, monitoring the inflammatory response and trajectory of the septic patient, and assessing the risk for ICU delirium. Such simulations would allow clinicians to compare different approaches or therapeutical interventions and choose the most beneficial strategy for the patient, limiting the complications related to each treatment.

Despite their significant promise, digital twin technologies encounter several challenges regarding data integration from heterogenous sources, standardized data acquisition, interoperability and transparent interpretability. Nevertheless, implementation of AI tools and digital twin models require rigorous validation, prospective data, ethical governance and clinical accountability. The future of AI lays in the perspective of a transition from artificial intelligence to augmented clinical intelligence.

## 4. Limitations

AI-tools and machine learning technologies have evolved to potential clinical support tools, with great perspectives for future implementation, but currently with limited use in clinical practice. This narrative review has several limitations. It highlights the aspects found in current literature that is mainly based on retrospective studies. Evidence found in the current literature vary, therefore creating selection bias and differences between centers and the quality of data. Taken together, along with the lack of real-time testing, current findings require caution in interpretation and future prospective research is needed to address these limitations of the Smart-ICU concept.

## 5. Conclusions

Critical illness is characterized by complex interactions between multiple physiological systems. Throughout this review, we summarized the potential of artificial intelligence in supporting earlier recognition of deterioration and more individualized bedside decision-making, by integration of multimodal physiological, laboratory, and clinical data into a dynamic representation of the patient’s biological trajectory, acknowledging current limitations regarding the lack of prospective studies and clinical implementation validation studies.

Biology remains the foundation. Artificial intelligence extends our analytical capabilities. The physician gives both clinical meaning. The future of AI tools in intensive care is based on dynamic integration of physiological and pathological changes and clinical context. The most relevant applications of AI-tools in ICU are concerning sepsis, respiratory and hemodynamic deterioration, delirium—both as early recognition and prognostic tools. Currently, the main limitations are prospective clinical validation and implementation into clinical practice.

## Figures and Tables

**Table 1:** Data from the literature regarding AI predictive models, clinical applications, evaluation metrics and current limitations.

Study	Model/ Tool	Clinical Application	Sample Size	Outcomes	Sensitivity	Specificity	CI	AUROC/AUPRC	Limitations
Mollura et al. [12]	closed-loop AI model	Sepsis detection using ECG and ABP continuous waveforms	Retrospective data from MIMIC-III database	Identifying sepsis within the first hour of admission	0.86	0.8	95% CI: 0.79–0.99	AUROC = 0.92, AUPRC = 0.90	Retrospective single-center design, lack of external prospective validation
Cherifa et al. [17]	MTL-PLD	Avoid hypotensive episodes and end-organ hypoperfusion	MIMIC-III database and French hospital cohort for external validation	Prediction of MAP and HR 60 min before			95% CI: 0.692–0.794		Lack of prospective validation
Chiang et al. [19]	TvHEWS	Prediction of hemodynamic instability in ICU	retrospective data -VGHTPE 2010 cohort;	Prediction up to 7 h before intervention	0.94	0.77		AUROC 0.93	Need of further external prospective validation
			prospective data—VGHTPE 2022 cohort;	Prediction up to 8.6 h before intervention	0.74	0.76		AUROC 0.92	
			external validation—MIMIC IV cohort	Prediction up to 21 h before intervention	0.72	0.37		AUROC 0.82	
Rahman et al. [20]	HSI (Abstain-Boost model)	Prediction of future hemodynamic status and interventions	364 ICUs in the USA; MIMIC-III database for external validation	Prediction of hemodynamic interventions 1 h before	0.92	0.52		AUC 0.82 for all hemodynamic interventions; AUC 0.88 for vasopressor initiation	Retrospective observational study
Shashikumar et al. [21]	Deep learning algorithm	Prediction of future need for mechanical ventilation in hospitalized patient	>30.000 ICU patients	24 h prediction of mechanical ventilation requirement	0.84	0.83		AUC 0.895; AUC 0.918–0.943 for COVID patients	Need for further external validation
Kulkarni et al. [22]	DenseNet121—deep learning using CNN architecture	Prediction of need for mechanical ventilation based on thoracic X-ray images in COVID patients	663 X-ray images from 528 patients	Prediction of mechanical ventilation 3 days before intubation	0.86	0.84		not reported	Single-center dataset, developed specific for COVID patients, lack of validation in non-COVID patients
Nemanti et al. [26]	ML-AISE	Early sepsis prediction	>31.000 ICU admission; development cohort and external validation cohort	Prediction of sepsis 4 h before clinical recognision	0.85	0.67	95% CI: 0.80–0.86	AUROC 0.83	Retrospective observational design. Further external validation needed
Wong et al. [27]	ESM	External validation of ESM	27,697 patients	Evaluating the ESM’s performance in prediction of the onset of sepsis	0.33	0.86	95% CI, 0.62–0.64	AUROC 0.63	Single-center study
Adams et al. [28]	TREWS	Improving patient outcome with TREWS monitoring and alerting	590,736 patients monitored, 6877 patients with sepsis before initiation of antimicrobial therapy	18.7% reduction in in-hospital mortality when alarms were confirmed within 3 h	Not reported	Not reported	CI 9.4–27.0%	Not reported	Observational implementation study and did not report accuracy metrics
Park et al. [37]	Random Forest model	delirium predictive model	5478 records–development cohort, 4438 records–temporal validation, 670 patients–external validation	Prediction of delirium before clinical diagnosis in ICU patients			Internal validation: CI 0.83, external validation: CI 0.84, temporal validation: CI 0.73	Internal validation: AUROC of 0.82, Temporal validation: AUROC of 0.73; External validation AUROC of 0.82	Retrospective design, requires further prospective external validation

ABP—arterial blood pressure; AI—artificial intelligence; AISE—Artificial Intelligence Sepsis Expert; AUROC—Area Under the Receiver Operating Characteristic curve; AUPRC—Area Under the Precision-Recall Curve; CI—Confidence Interval; CNN—convolutional neural network; ECG—electrocardiography; ESM—Epic Sepsis Model; HR—heart rate; HSI—hemodynamic stability index; ICU—intensive care unit; MAP—mean arterial pressure; ML—machine learning; MTL-PDL—Multi-task Learning Physiological Deep Learner; TREWS—Targeted Real-time Early Warning System; TvHEWS—Time-varying Hemodynamic Early Warning Score.

## Data Availability

No new data were created or analyzed in this study. Data sharing is not applicable to this article.

## Abbreviations

The following abbreviations are used in this manuscript:

ABP	Arterial Blood Pressure
AI	Artificial Intelligence
AISE	Artificial Intelligence Sepsis Expert
APACHE	Acute Physiology and Chronic Health Evaluation
ARDS	Acute Respiratory Distress Syndrome
AUC	Area Under the Curve
AUROC	Area Under the Receiver Operating Characteristic curve
AUPRC	Area Under the Precision-Recall Curve
CAM-ICU	Confusion Assessment Method for the Intensive Care Unit
CI	Confidence Interval
CNN	Convolutional Neural Network
CO2	Carbon Dioxide
ECG	Electrocardiogram
EEG	Electroencephalography
EHR	Electronic Health Record
ESM	Epic Sepsis Model
GFAP	Glial Fibrillary Acidic Protein
HR	Heart rate
HSI	Hemodynamic Stability Index
HRV	Heart Rate Variability
ICU	Intensive Care Unit
LV-GLS	Left Ventricular Global Longitudinal Strain
MAP	Mean Arterial Pressure
MEWS	Modified Early Warning Score
ML	Machine Learning
MTL	Multi-Task Learning
MTL-PDL	Multi-task Learning Physiological Deep Learner
MPM	Mortality Probability Model
NEWS	National Early Warning Score
NIRS	Near-Infrared Spectroscopy
PPG	Photoplethysmography
PvaCO2	Venous-to-Arterial Carbon Dioxide Partial Pressure Difference
Q-EEG	Quantitative Electroencephalography
ROX	Respiratory rate–Oxygenation Index
SAPS	Simplified Acute Physiology Score
SICD	Sepsis-Induced Cardiac Dysfunction
SOFA	Sequential Organ Failure Assessment
SpO2	Peripheral Oxygen Saturation
ST	ST segment
TREWS	Targeted Real-time Early Warning System
TvHEWS	Time-varying Hemodynamic Early Warning Score
